# Definition of Outcome-Based Prostate-Specific Antigen (PSA) Thresholds for Advanced Prostate Cancer Risk Prediction

**DOI:** 10.3390/cancers13143381

**Published:** 2021-07-06

**Authors:** Simona Ferraro, Marco Bussetti, Niccolò Bassani, Roberta Simona Rossi, Giacomo Piero Incarbone, Filippo Bianchi, Marco Maggioni, Letterio Runza, Ferruccio Ceriotti, Mauro Panteghini

**Affiliations:** 1 Unità Operativa Patologia Clinica, ASST Fatebenefratelli-Sacco, Ospedale ‘Luigi Sacco’, Via GB Grassi 74, 20157 Milano, Italy; marco.bussetti@unimi.it (M.B.); mauro.panteghini@unimi.it (M.P.); 2 Statistical Consultant, Flat 5 Hazel Court Avenue, Hitchin SG4 9SJ, UK; niccolo.bassani@gmail.com; 3 Unità Operativa Anatomia Patologica, ASST Fatebenefratelli-Sacco, Ospedale ‘Luigi Sacco’, Via GB Grassi 74, 20157 Milano, Italy; rossi.roberta@asst-fbf-sacco.it (R.S.R.); filippo.bianchi@asst-fbf-sacco.it (F.B.); 4 Urologia, ASST Fatebenefratelli-Sacco, Ospedale ‘Luigi Sacco’, Via GB Grassi 74, 20157 Milano, Italy; incarbone.giacomo@asst-fbf-sacco.it; 5 Unità Operativa Anatomia Patologica, Fondazione IRCCS Ca’ Granda Ospedale Maggiore Policlinico Via F. Sforza 35, 20122 Milano, Italy; marco.maggioni@policlinico.mi.it (M.M.); letterio.runza@policlinico.mi.it (L.R.); 6 Laboratorio Analisi, Fondazione IRCCS Ca’ Granda Ospedale Maggiore Policlinico Via F. Sforza 35, 20122 Milano, Italy; ferruccio.ceriotti@policlinico.mi.it; 7 Dipartimento di Scienze Biomediche e Cliniche ‘Luigi Sacco’, Università Degli Studi di Milano, 20157 Milano, Italy

**Keywords:** calibration, risk prediction, prostate cancer, immunoassay, inflammation

## Abstract

**Simple Summary:**

In this study, we used a well calibrated risk prediction model to define prostate-specific antigen (PSA) thresholds for identifying or excluding advanced prostate cancer (PCa) as an aid to personalize management of the diagnostic workup. PSA concentrations ≤ 4.1 (<65 years old) and ≤3.7 μg/L (≥65 years old) excluded an advanced PCa in patients without glandular inflammation, while PSA > 5.7 (<65) and >6.1 μg/L (≥65) suggested a biopsy referral. In the presence of glandular inflammation, PSA does not provide a valid estimate for risk of advanced cancer since the marker variability is higher and the pre-test probability of PCa is low in this group. The proposed PSA thresholds may allow an individualized approach to the diagnostic workup, assisting patients in making an informed decision. However, patients with asymptomatic prostatitis cannot benefit from the use of this model since they cannot be pre-biopsy identified.

**Abstract:**

We defined prostate-specific antigen (PSA) thresholds from a well calibrated risk prediction model for identifying and excluding advanced prostate cancer (PCa). We retrieved 902 biopsied patients with a pre-biopsy PSA determination (Roche assay). A logistic regression model predictive for PCa including the main effects [i.e., PSA, age, histological evidence of glandular inflammation (GI)] was built after testing the accuracy by calibration plots and Hosmer-Lemeshow test for goodness of fit. PSA thresholds were derived by assuming a diagnostic sensitivity of 95% (rule-out) and 80% (rule-in) for overall and advanced/poorly differentiated PCa. In patients without GI, serum PSA concentrations ≤ 4.1 (<65 years old) and ≤3.7 μg/L (≥65 years old) excluded an advanced PCa (defined as Gleason score ≥ 7 at biopsy), with a negative predictive value of 95.1% [95% confidence interval (CI): 83.0–98.7] and 88.8% (CI: 80.2–93.9), respectively, while PSA > 5.7 (<65) and >6.1 μg/L (≥65) should address biopsy referral. In presence of GI, PSA did not provide a valid estimate for risk of advanced cancer because of its higher variability and the low pre-test probability of PCa. The proposed PSA thresholds may support biopsy decision except for patients with asymptomatic prostatitis who cannot be pre-biopsy identified.

## 1. Introduction

Recently released clinical practice guidelines (CPGs) no longer recommend decision thresholds of prostate-specific antigen (PSA) for the early detection of prostate cancer (PCa) to rule in patients for prostate biopsy referral [1,2,3]. Rather the expert panels have endorsed the stratification of PCa risk according to individual PSA values and age, aiming to offer biopsy to patients at increased risk of high-grade disease, to promote active surveillance of slow-growing PCa, and to run effective longitudinal PSA retesting programs [1,4]. Accordingly, CPGs try to adapt the time of repetition of PSA testing to the estimated individual risk, with the result of heterogeneous recommendations [1]. For instance, the American Cancer Society indicated a yearly PSA retesting for all patients with a value ≥ 2.5 µg/L at baseline, whereas other CPGs lowered the threshold to 1.0 µg/L, restricted the monitoring to men aged 55–69 years, and/or extended the time interval for PSA rescreening to 2–4 years [1,2,3,5].

The main challenge remains to use PSA results to identify patients for prostate biopsy referral who most likely will benefit from an intervention defined by a shared decision made between the patients and the care provider [4,6]. A wide spectrum of pre-biopsy risk nomograms has been proposed as an aid in decision with the aim to maximize the diagnostic specificity, but the overall poor predictive capability resulting from the validation phases has conditioned the recommendation of their use [7,8,9]. On the other hand, several methodological issues have been identified within studies developing pre-biopsy risk models, such as low numbers of biopsy core that were examined, presence of verification bias in studies including men from screening programs, lack of calibration of predictive models, and/or use of different PSA assays as an additional cause of miscalibration and source of intra-study heterogeneity [1,9,10,11,12]. Some experts have asked how much accurate the estimate of the risk predicted at the individual level is, underlining the burden of unnecessary biopsies and the rate of missed aggressive cancer [13]. These drawbacks stress the use of a more rigorous research methodology, as well as the need to tune risk models to the current clinical goals in order to pragmatically fulfill the redesigned screening strategies. In other words, the predicted risk should inform an individual on the probability to exclude or detect an aggressive PCa, with an acceptable accuracy of recommending biopsy referral. 

There is an unmet need for the use of PSA testing to prevent overdiagnosis, overtreatment, and unnecessary invasive procedures. The clinical goals for the use of PSA test in decision making should be better defined. Studies in literature which have evaluated the performance of the risk models, recommended values for diagnostic sensitivity ranging between 90% and 95%, while the ratio of one cancer detected for every three performed biopsies [corresponding to a positive predictive value (PPV) of 33.3%] was considered acceptable [9,14]. On the other hand, the estimate and the reliability of predictive values are strongly dependent on the rate of PCa incidence in the referral population [15,16], so that epidemiological data should be kept in mind to ultimately transfer affordable risk thresholds into the clinical practice. Available data indicate a 1:4 ratio between the annual incidence of PCa in men aged 50–64 years vs. the 65-over 85 years group, with an exponential growth between 50 and 64 years and a plateau after 65 years [17]. Furthermore, to exploit the full potential of PSA-based risk models for biopsy referral, the outcome should shift from the detection of PCa of any grade to advanced grade PCa only, given the low lethality and the questionable benefit of treating men with low-grade cancer [4,6,13]. By considering the highlighted issues, in this study we defined PSA thresholds using a well calibrated risk prediction model for identifying or excluding advanced PCa, as an aid in personalized management of the diagnostic workup of redesigned screening programs. 

## 2. Results

### 2.1. Characteristics of Studied Patients

**Figure 1:**
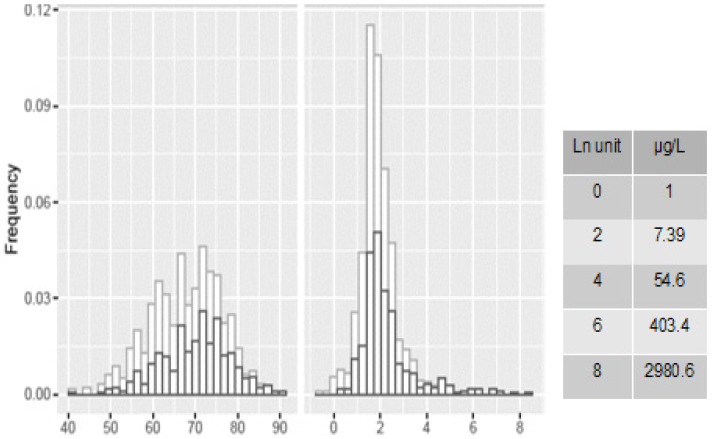
Distribution of age (left) and ln PSA concentrations (right) in patients with (black histograms) and without (grey histograms) prostatic cancer.

The main features of the studied case series are reported in Table 1. Out of 902 patients, 415 (46.0%) had PCa. Patients with PCa were older and had higher PSA values than those without the disease. Both age and serum PSA concentrations were significantly increased in advanced stages of PCa, with ~80% of patients with advanced/poorly differentiated disease aged over 65 years. Although the distribution of age was symmetrical (mean, 67.9 years), the distribution of PSA values was skewed with a heavy tail on the right end. Accordingly, PSA concentrations followed natural logarithmic transformation (ln) to generate a near-normal distribution. Figure 1 and Figure S1 in the online Data Supplement display the distributions of age and ln PSA in patients with and without PCa, and with or without histological evidence of glandular inflammation, respectively. The presence of glandular inflammation markedly prevailed in patients free of cancer vs. those diagnosed with PCa (41.7% vs. 8.2%, *p* < 0.001), and far lower in advanced (4.9%) and poorly differentiated PCa (1.6%). In cancer-free patients, the presence of inflammation significantly increased PSA concentrations [6.64 μg/L (4.6–11.1) vs. 5.2 μg/L (3.7–7.3); *p* < 0.0001]. 

### 2.2. Predictive Models 

Logistic regression models predictive for PCa which showed the best goodness of fit (Akaike information criterion, AIC = 977.1) and predictive ability (area under the ROC curve, AUC = 0.79, CI: 0.76–0.82) included the main effects, i.e., PSA on ln scale, age, histological evidence of glandular inflammation, as covariates, and the PSA by age interaction. Calibration plots on overall case series using as outcome the diagnosis of PCa of any grade, Gleason score ≥ 7 and ISUP grade ≥ 3 PCa did not show a systematic pattern of over- or underestimation (Figure S2). However, some fluctuations around the diagonal line occurred, in particular for fitted predicted probability values < 0.4 with Gleason score ≥ 7 and ISUP grade ≥ 3 PCa outcomes, and <0.8 with PCa of any grade as outcome. Therefore, the Hosmer-Lemeshow goodness of fit test was considered using four groups to split the predicted probabilities [number of groups = number of covariates (*p* = 3) + 1]. The test produced non-significant *p* values for all models (0.06 for any grade tumors, 0.78 for Gleason score ≥ 7, and 0.85 for ISUP grade ≥ 3 PCa, respectively), suggesting there be no difference between predicted and observed probabilities. By considering subgroups partitioned according to the age threshold of 65 years, well calibrated models were observed in patients without evidence of glandular inflammation (Figures S3 and S4).

Derived logistic regression equations estimated that an absolute PSA increase of 1 ln unit (corresponding to 2.72 µg/L) implied an age-adjusted odds ratio (OR) of 2.72 (CI: 2.17–3.49), 5.3 (CI: 3.95–7.51), and 7.00 (CI: 4.93–10.54) in harboring any grade, advanced, and poorly differentiated PCa, respectively. Table 2 shows the individual probabilities [estimated according to the formula: 1/(1 + *e*^−log odds^) [18]] of harboring a PCa of any grade and of aggressive grade by assuming a PSA of 4.0 μg/L.

### 2.3. PSA Thresholds for Decision Making

The fitted probabilities of overall and advanced PCa (according to Gleason and ISUP grading) vs. PSA values (ln scale), partitioned according to age threshold of 65 years and possible presence of glandular inflammation were visually inspected (Figures S5 and S6). In patients with histological evidence of glandular inflammation, the low PCa prevalence was a major drawback in the definition of PSA risk thresholds for advanced cancer and biopsy referral. Our data only provide the possibility to make some inference on the ability of PSA testing to exclude PCa in patients ≥ 65 years old with evidence of glandular inflammation: in this subgroup, a PSA value < 8.3 µg/L ruled out the presence of PCa of any grade with a negative predictive value (NPV) of 98.6% (CI: 90.8–99.8). 

Considering the 381 PCa patients without histological evidence of glandular inflammation, we were able to estimate for each partition, by setting the diagnostic sensitivity at 95% (rule out) and 80% (rule in), the corresponding PSA thresholds, together with the estimated sensitivity, specificity, and predictive values (Table 3). In patients < 65 years old, a PSA value > 5.7 µg/L was associated to a PPV of 35.9%, corresponding to approximately one advanced cancer detected every three performed biopsies. PSA values ≤ 4.1 µg/L and ≤4.9 µg/L ruled out advanced (Gleason score ≥ 7) and poorly differentiated (ISUP grade ≥ 3) PCa, with a NPV of 95.1% and 97.5%, respectively. In patients ≥ 65 years old, PSA threshold values of >5.3 µg/L (Gleason score ≥ 7) and >6.1 µg/L (ISUP grade ≥ 3) can be adopted for biopsy referral, giving a PPV slightly higher than 50%, indicating a rate of ~1:2 between detected cancers and performed biopsies. On the other hand, PSA results < 3.7 µg/L excluded the presence of an advanced PCa, with a NPV of 88.8%.

### 2.4. Developing a PSA-Based Diagnostic Workup

**Figure 2:**
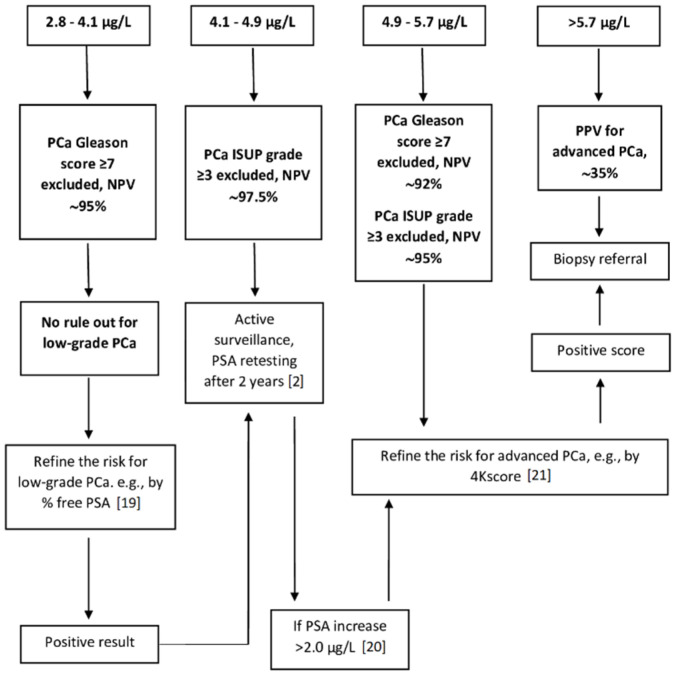
PSA—based diagnostic workup for patients < 65 years old, with no evidence of glandular inflammation on histology. This workflow includes recommendations on second level approaches inferred from the available literature (references reported in parentheses). The above values represent pre-biopsy serum PSA concentrations. In black, information derived by this study. Note: Recommended active surveillance implies PSA retesting after two years and it is stopped if the risk of advanced cancer increases according to an absolute PSA increase over 2 µg/L. It is reasonable to endorse the PSA retesting interval recommended by the American Urological Association [2]. PCa, prostatic cancer; NPV, negative predictive value; ISUP, International Society of Urological Pathology; PPV, positive predictive value; 4Kscore, 4-kallikrein panel score.

**Figure 3:**
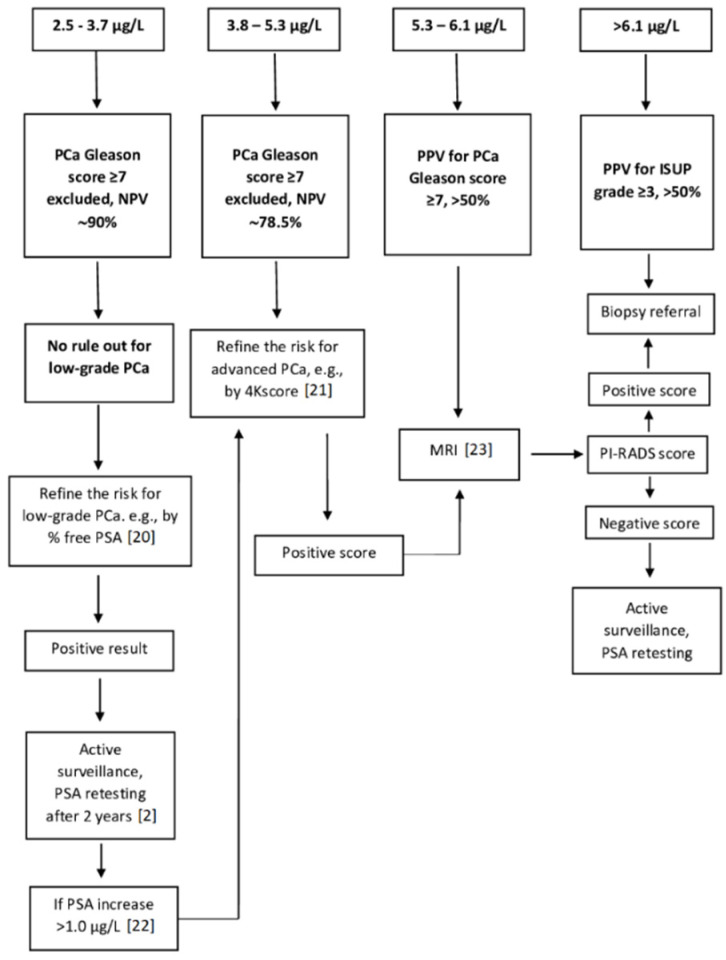
PSA—based diagnostic workup for patients ≥ 65 years old, asymptomatic for glandular inflammation, including recommendations on second level approaches inferred from the available literature (references reported in parentheses). The above values represent pre-biopsy serum PSA concentrations. In black, information derived by this study. Note: Recommended active surveillance implies PSA retesting after two years and it is stopped if the risk of advanced cancer increases according to an absolute PSA increase over 1 µg/L. It is reasonable to endorse the PSA retesting interval recommended by the American Urological Association [2]. PCa, prostatic cancer; NPV, negative predictive value; ISUP, International Society of Urological Pathology; PPV, positive predictive value; 4Kscore, 4-kallikrein panel score; MRI, multiparametric magnetic resonance imaging; PD-RAS, Prostate Imaging-Reporting and Data System.

Figure 2 and Figure 3 display the PSA-based diagnostic workup for patients aged <65 and ≥65 years without evidence of prostate inflammation, determined based on the evidence obtained in our study, and including some additional second level tests, whose diagnostic performance aiding to refine the individual risk was demonstrated by the literature [19,20,21,22,23].

## 3. Discussion

Current CPGs for early detection of PCa advise for a more selective use of PSA testing in the context of “shared decision-making”, and promote individualized risk-adapted approaches to screening, biopsy referral, and treatment [1,4]. It is currently recommended to estimate the individual PSA-based odds of PCa to address the rescreening interval, active surveillance, and biopsy referral, seeking for a reduction of harms associated with the procedure invasiveness, overdiagnosis, and overtreatment of low-risk disease. Several meta-analyses have reported the contribution of risk models to improve the discriminative capability of PSA for PCa [7,8,9], and authoritative voices have claimed that the risk-benefit ratio is enhanced when the estimate of individual risk is unbiased and precise, the rule in capability to detect advanced stages is high, and there is a considerable decrease of undue biopsies [13,24]. To shift the harm-benefit balance, risk models should: (a) theoretically include variables easy to collect without requiring further expensive/invasive tests (e.g., assessment of prostate volume), (b) be well calibrated, showing a good agreement between the individual patient’s estimated risk and the true risks, and (c) consider PSA thresholds in relation to a specific age range, since the predictive ability differs across age groups [13,25,26,27]. Our well calibrated model shows that PSA concentrations, histological evidence of prostatic inflammation and older age independently contributed to the estimation of the individual risk. 

It should be noted that in the majority of cases the histological presence of glandular inflammation confirms the clinical diagnosis of symptomatic prostatitis, the latter having higher prevalence with respect to the asymptomatic inflammation in patients over 60 years of age [28,29]. Histology contributes to the definition of an (asymptomatic) prostatitis in only ~30% of biopsies performed in a screened population and more rarely (18% of diagnoses) are associated with a PSA increase over 4.0 µg/L [29,30,31,32]. This is an important point to consider since the proposed risk model may generally run in a pre-biopsy setting as an aid to recommend biopsy referral if symptomatic prostatitis is excluded, but also may aid in the post-biopsy setting when histology shows an asymptomatic inflammation to prevent further investigations.

Age partitioning according to a single threshold of 65 years permits a well-powered evaluation in the subgroups, with an overall sample size and PCa prevalence in agreement with other large pre-biopsy cohorts [8]. This also takes into consideration that the ratio between the number of PCa cases and variables included in the model should not be <10:1 [33]. This age threshold is supported by the evidence that the bulk of data on pre-biopsy cohorts is retrieved from men with average ages falling in the 61–70 years range and that the current life expectancy in Western countries is ~22 years for 65-year-old men [8]. Furthermore, epidemiological data indicates that the risk of PCa follows an exponential trend after 50 years of age (113 cases/100,000/year in the range of 50–54 years vs. 495/100,000/year in the range of 60–64 years, respectively), reaching a plateau in individuals over 65 years [17].

Some interesting information can be derived from our study. First, we confirmed that, even in patients < 65 years old, the selection of a PSA threshold of 4.0 µg/L for biopsy referral implies an unfavorable risk-benefit ratio, leading to an overdiagnosis of low-grade tumors (individual probability of advanced PCa ~10%) [34]. In this group of patients, the poor cost-benefit ratio of PSA (re)screening for men with PSA values < 4.0 µg/L is conditioned by the very low risk of advanced PCa in this range of values and by the amount of PSA increase needed to significantly influence the ratio at the individual level, with a progression towards advanced clinical stages or a change in tumor grade assignment. Our evidence is aligned with other research data reporting that only ≤20% of men undergoing screening with PSA in the range 2.6–4.0 µg/L harbored a Gleason score ≥ 7 PCa [14,19,35]. On the other hand, the evidence suggests that rescreening men aged <65 years with PSA at baseline > 4.0 µg/L for active surveillance of low-grade disease may have a favorable risk-benefit balance if performed biennially [2], in particular considering: (a) the slow growth of PCa of any histological grade, and (b) the reference change value of ~20% useful to identify intraindividual PSA changes overtime significantly turning into clinically relevance [25,36,37]. Taking that into account, in our study, PSA values between 4.1 and 4.9 µg/L consistently excluded aggressive PCa (NPV, 97.5%), therefore suggesting to place these patients under active surveillance with a biennial PSA retesting (Figure 2). A PSA increase > 2.0 µg/L for baseline concentrations > 4.0 µg/L may significantly enhance the identification of aggressive PCa [20,38]. In patients with PSA values between 4.9 and 5.7 µg/L, in which the PSA rule-out power for advanced PCa is decreasing, a second level test, such as the 4-kallikrein panel score (4Kscore), may aid in the identification of advanced cancer risk, assuring a PPV > 95% [21]. Finally, it sounds reasonable in this population with a long-life expectancy to consider the PSA level > 5.7 µg/L for biopsy referral, since that at this marker concentration the ratio of one advanced cancer detected every 3 biopsies performed is fulfilled. 

In patients ≥ 65 years old, the rule-out power of PSA for advanced PCa, even in absence of glandular inflammation, is significantly reduced and the estimate of free PSA percentage may help to increase the NPV in patients with PSA concentrations ≤ 3.7 µg/L [19]. It is noteworthy that the predictive power of PSA testing in men > 62 years with a baseline PSA ≤ 4.0 µg/L changes slowly across subsequent rescreening rounds, being associated to an average OR of only 2.1 for advanced PCa, when the value increased of 1.0 µg/L [22]. For patients with higher PSA values, the use of a second level test, such as the 4Kscore, able to increase the PPV and the addition of multiparametric magnetic resonance imaging may refine the risk for advanced cancer and optimize biopsy referral (Figure 2) [21,23,39]. In patients with PSA values > 6.1 µg/L, biopsy referral should be recommended. 

It should be clarified that in the proposed diagnostic workup we recommend the use of second level investigations, such as free PSA percentage or 4Kscore, for patients falling within defined PSA ranges just considering their predictive contribution added to the baseline PSA values [19,21]. Notably, the use of 4Kscore is conditioned by the difficult availability of the test; alternatively, the prostate health index may be considered in those laboratories measuring PSA by Beckman Coulter assay [21]. 

In our study, we were unable to define PSA risk thresholds for advanced cancer and biopsy referral in patients displaying glandular inflammation. This was due to the known low prevalence of PCa in this subgroup of patients and to the variable effect of the inflammatory status on the increase of PSA concentrations in serum [21,32,36]. This could not be circumvented by collapsing these partitions, since ROC curves built on the full model may consequently deviate from the monotonic function. In this situation, similar PSA results might be erroneously associated with significantly different predictive values. This risk is increased for biomarkers like PSA characterized by high inter-individual biological variability, which conditions *per se* the reliability of cut-off values [40]. The setup of risk prediction models on such pre-biopsy cohorts, although excluding the verification bias, may provide an overestimation of PSA predictive ability. In clinical practice, we may overcome this issue regarding whether the risk algorithm is addressed to patients with a considerable pre-test probability, according to the presence at baseline of known risk factors (i.e., ethnicity, family history, older age, positive digital rectal examination, and transrectal ultrasonography prostate volume), which however we were unable to completely retrieve in our evaluated patients [1]. 

A major limitation of our study is represented by the difficulty to clinically define the presence of glandular inflammation, a significant confounder of PSA interpretation, in a pre-biopsy setting. The majority of patients with prostatitis are symptomatic and may be diagnosed even if symptoms, such as dysuria, may be nonspecific. However, asymptomatic prostatitis may be occasionally detected in patients undergoing biopsy [29]. Therefore, studies conducted to identify serum biomarkers that may help to detect prostatic inflammation by adding their measurements to PSA in a pre-biopsy setting are warranted. If prostatitis is suspected clinically, administration of an antibiotic therapy before PSA testing is recommended. Previous studies showed that antibiotics may help avoid biopsy in selected patients whose PSA elevation was likely due to prostatitis [41]. A further limitation may be that PSA elevation may enter in the clinical criteria for biopsy referral and an overestimation of its predictive value may be suspected. However, this type of bias is likely to occur when the index test enters in the clinical criteria for determining the diagnostic outcome and this is not the case here since the diagnosis of PCa was independent of PSA being based on histological criteria.

## 4. Materials and Methods 

### 4.1. Patients, Histological Diagnosis, and PSA Determination

We retrospectively retrieved a continuous case series of 902 patients, tested for total PSA in two academic hospitals in Milan from 2014 to 2020, before performing a transrectal ultrasound guided prostate biopsy.

The criteria used to recommend biopsy were the presence of known risk factors (i.e., family history and older age) associated to dubious or positive digital rectal examination, and/or to an increased transrectal ultrasonography prostate volume, and/or to a free PSA < 20%.

Patients with a previous biopsy or an established diagnosis of PCa were excluded. Disease status (i.e., evidence/no evidence of PCa and of prostatic inflammation) was determined histologically for all patients by prostate biopsy on >12 cores. Gleason score and the International Society of Urological Pathology (ISUP) grade were used for PCa grading, the latter being now recommended to fulfill the updated CPGs [42]. PCa with a Gleason score ≥ 7 and an ISUP grade ≥ 3 have been both associated to a worse prognosis, but ISUP grading allows to split the Gleason score 7 patterns, including the prognostically different 3 + 4 and 4 + 3 groups, in ISUP grade 2 and 3, respectively [43]. The group 3 + 4 (ISUP grade 2) mostly includes well differentiated cancers, whereas the group 4 + 3 (ISUP grade 3) mostly includes poorly differentiated cancers [43]. 

The presence of prostatic inflammation was based on histological criteria and defined by an evident, not random presence of a granulocytic infiltrate in the histologic sections [44]. The characterization of the inflammatory pattern contributes to the clinical diagnosis of prostatitis syndromes classified into four groups according to National Institute of Health (NIH) criteria [29]. It has been reported that 90% of symptomatic patients have inflammatory chronic prostatitis/chronic pelvic pain syndrome, while the remaining having asymptomatic prostatitis are defined on the basis of the histological evidence of an inflammatory pattern in subjects undergoing biopsy because of an occasional PSA elevation [29]. In our case series, patients with histological evidence of glandular inflammation were however not partitioned according to NIH categories or dichotomized according to the presence/absence of symptoms.

PSA measurements were carried out using an electrochemiluminescence immunoassay marketed by Roche Diagnostics, whose characteristics have been previously described in detail [36]. PSA for patients enrolled until 2017 were assayed on a Cobas e601 platform, while in the last three years the newly released Cobas e801 analyzer was employed. Results from the two systems were nicely aligned [36]. The review board of our institution approved the study, carried out according to the Helsinki Declaration of 1975, as revised in 1996. 

### 4.2. Statistical Analysis

The required sample size was estimated according to a simulation based on previously published data [36], by setting µ1 = 6.7 μg/L and µ2 = 5.5 μg/L as means of distribution of PSA values in patients with and without PCa in the referral population, a SD σ = 3.5 μg/L, one-sided test option, α = 0.05, and a power of 0.80 [45]. By considering a maximum of 5 theoretical partitions of clinical interest, a final sample size of 800 patients may prompt to a well powered investigation, as also confirmed by the available evidence [15].

Data were reported as percentages for categorical variables and median with interquartile range for quantitative variables. Differences between variables in different categories were assessed by applying chi-squared test (categorical) and Mann-Whitney rank-sum test (quantitative). Logistic regression models predictive for PCa including the main effects (PSA value, age, evidence of inflammatory pattern at histology) were separately built and the one with the greatest goodness of fit [estimated by Akaike information criterion (AIC)] and the highest predictive ability [estimated by the area under the ROC curve (AUC)] was selected. The accuracy of the final model was assessed graphically by inspecting the calibration plots [using locally weighted scatterplot smoothing (LOWESS), with a span of 0.1] and formally by using the Hosmer-Lemeshow test for goodness of fit (splitting the predicted probabilities in four groups) [8,13]. Sensitivity, specificity, and predictive values associated with defined PSA risk thresholds were derived. 95% confidence intervals (CI) for sensitivity and specificity were estimated by Clopper-Pearson exact method, while CI for predictive values were obtained using a Wald-type interval on a logit scale. Patient age was partitioned using the threshold of 65 years according to the actual prevalence of PCa [17]. All statistical analyses were performed using R software, version 3.6.3 (R Foundation for Statistical Computing, Vienna, Austria

## 5. Conclusions

In conclusion, the proposed PSA-based risk prediction approach may allow individualization of the diagnostic workup and balance the prediction of advanced PCa and biopsy referral between harms and benefits, thus permitting patients to make an informed decision. The greatest limitation remains the clinical difficulty to exclude the presence of asymptomatic prostatitis in a pre-biopsy setting, which however should rarely occur in patients with a significant PSA increase.

PSA is an inexpensive diagnostic tool, but it may actually trigger excessive costs by increasing the second-level tests workload and affect the individual harm-benefit balance if results are not appropriately managed [46,47]. We propose that future clinical trials should be designed prospectively to validate the proposed strategy. Furthermore, we are unaware if our PSA risk thresholds should be considered method-dependent or may be widely applied, given that the harmonization among marketed PSA measuring systems may necessitate further improvement [10].

## Figures and Tables

**Table 1:** Main characteristics of the studied case series.

Patients	Age, Years ^a^	No. ≥ 65 Years Old	PSA, µg/L ^a^	Patients with Histological Evidence of Glandular Inflammation
Overall (n = 902)	69 (62–74)	594 (65.9%)	6.1 (4.5–9.9)	237 (26.3%)
Non PCa (n = 487; 54.0%)	66 (60–73)	278 (57.1%)	5.6 (4.1–8.2)	203 (41.7%)
PCa (n = 415; 46.0%)	71 (65–76) ^b^	316 (76.1%)	7.3 (5.1–12.3) ^b^	34 (8.2%)
PCa with Gleason score < 7 (n = 168; 40.5%)	70 (63–74)	118 (70.2%)	5.6 (4.2–7.4)	22 (13.1%)
PCa with Gleason score ≥ 7 (n = 247; 59.5%)	71 (66–77) ^c^	197 (79.8%)	9.8 (6.1–23.4) ^c^	12 (4.9%)
PCa with ISUP grade < 3 (n = 231; 55.7%)	70 (63–75)	164 (71.0%)	5.8 (4.4–8.2)	31 (13.4%)
PCa with ISUP grade ≥ 3 (n = 184; 44.3%)	72 (67–77) ^d^	151 (82.1%)	11.6 (7.1–33.6) ^d^	3 (1.6%)

PCa, prostatic carcinoma; ISUP, International Society of Urological Pathology. ^a^ Median and interquartile range. ^b^ *p* < 0.001 vs. non PCa. ^c^ *p* < 0.001 vs. Gleason score < 7. ^d^ *p* < 0.001 vs. ISUP < 3.

**Table 2:** Simulation of the probability of harboring a prostatic cancer (PCa) of any grade, advanced (Gleason score ≥ 7), and poorly differentiated grade (ISUP ≥ 3) for an individual with a baseline PSA value of 4.0 μg/L.

Outcome	Equation ^a^	Histological Evidence of Glandular Inflammation	Age, Years	Individual Probability ^b^ (95% CI)
PCa of any grade	Log odds = −2.1 + ln(PSA) + 0.80 age (≥65 years) − 2.50 inflammation (yes)	No	<65	33.5% (27.4–40.3)
			≥65	53.0% (47.3–58.3)
		Yes	<65	3.9% (2.3–6.3)
			≥65	8.4% (5.2–12.4)
Gleason score ≥ 7	Log odds = −4.5 + 1.68 ln(PSA) + 0.83 age (≥65 years) − 3.32 inflammation (yes)	No	<65	10.2% (7.1–14.7)
			≥65	19.5% (16.5–25.9)
		Yes	<65	0.4% (0.2–1.0)
			≥65	0.9% (0.4–2.1)
ISUP grade ≥ 3	Log odds = −5.7 + 1.96 ln(PSA) + 0.97 age (≥65 years) − 4.91 inflammation (yes)	No	<65	4.9% (2.9–7.9)
			≥65	11.9% (8.6–15.9)
		Yes	<65	0.04% (0.008–0.2)
			≥65	0.1% (0.02–0.5)

^a^*p* < 0.000001 for all coefficients in the equations. ^b^ Estimated according to the formula: 1/(1 + *e*^−log odds^).

**Table 3:** PSA risk thresholds and estimated diagnostic parameters derived by assuming sensitivities of 95% and 80% for overall and advanced/poorly differentiated prostatic cancer (PCa) by considering only patients without histological evidence of glandular inflammation.

Age Group	Outcome ^a^	Imposed Sensitivity	PSA Threshold, µg/L	Sensitivity, %	Specificity, %	PPV, %	NPV, %
<65 years	PCa of any grade (43.5%)	95%	2.8	94.6 (87.9–98.2)	6.6 (2.9–12.6)	43.8 (42.1–45.5)	61.5 (35.1–82.6)
		80%	4.2	79.6 (69.9–87.2)	26.4 (18.8–35.2)	45.4 (41.8–49.1)	62.7 (50.6–73.5)
	Gleason score ≥ 7 (21.5%)	95%	4.1	95.7 (85.2–99.5)	23.2 (17.1–30.3)	25.4 (23.5–27.4)	95.1 (83.0–98.7)
		80%	5.7	80.4 (66.1–90.6)	60.7 (52.9–68.1)	35.9 (30.7–41.5)	91.9 (86.2–95.4)
	ISUP grade ≥ 3 (14.9%)	95%	4.9	93.8 (79.2–99.2)	42.3 (35.0–49.8)	22.2 (19.7–25.0)	97.5 (90.9–99.3)
		80%	5.9	81.3 (63.6–92.8)	63.7 (56.3–70.7)	28.3 (23.4–33.7)	95.1 (90.3–97.6)
≥65 years	PCa of any grade (63.9%)	95%	2.5	94.8 (91.6–97.1)	17.2 (11.7–23.9)	66.9 (65.2–68.6)	65.1 (50.7–77.2)
		80%	4.7	79.9 (74.8–84.3)	40.5 (32.9–48.4)	70.3 (67.4–73.2)	53.2 (45.8–60.5)
	Gleason score ≥ 7 (41.5%)	95%	3.7	95.2 (91.1–97.8)	26.9 (21.6–32.7)	48.0 (46.0–50.0)	88.8 (80.2–93.9)
		80%	5.3	80.2 (73.8–85.7)	51.1 (44.9–57.3)	53.8 (50.2–57.3)	78.5 (72.8–83.3)
	ISUP grade ≥ 3 (32.8%)	95%	3.8	95.3 (90.5–98.1)	25.1 (20.3–30.4)	38.3 (36.6–40.1)	91.6 (83.7–95.8)
		80%	6.1	79.7 (72.3–85.9)	64.7 (59.0–70.1)	52.4 (48.1–56.7)	86.7 (82.4–90.1)

PPV, positive predictive value; NPV, negative predictive value. ^a^ Prevalence in parentheses.

## Data Availability

The data presented in this study are available in the article or supplementary files.

## Supplementary Materials

The following are available online at https://www.mdpi.com/article/10.3390/cancers13143381/s1, Figure S1: Distribution of age (left) and ln PSA concentrations (right) in patients patients with and without histological evidence of glandular inflammation, Figure S2: Calibration plots reporting the predicted probability of cancer of any grade (A), Gleason’s score ≥ 7 (B) and ISUP grade ≥ 3 (C) cancers by considering the overall case series. The grey zone represents the variability of the predicted values around the bisector. The cases are reported as black points, Figure S3: Calibration plots reporting the predicted probability of cancer of any grade (A), Gleason’s score ≥ 7 (B) and ISUP grade ≥ 3 (C) cancers by considering <65 years old patients with no histological evidence of glandular inflammation. The grey zone represents the variability of the predicted values around the bisector. The cases are reported as black points, Figure S4: Calibration plots reporting the predicted probability of cancer of any grade (A), Gleason’s score ≥ 7 (B) and ISUP grade ≥ 3 (C) cancers by considering ≥65 years old patients with no histological evidence of glandular inflammation. The grey zone represents the variability of the predicted values around the bisector. The cases are reported as black points, Figure S5: Fitted probabilities of prostatic cancer of any grade (A), with Gleason’s score ≥ 7 (B), and ISUP grade ≥ 3 (C) vs. PSA values (ln scale units) in the subgroup of patients with no histological evidence of glandular inflammation, partitioned according to age threshold of 65 years. Horizontal lines represent the 95% sensitivity thresholds. Figure S6: Fitted probabilities of prostatic cancer of any grade (A), with Gleason’s score ≥ 7 (B), and ISUP grade ≥ 3 (C) vs. PSA values (ln scale units) in the subgroup of patients with glandular inflammation, partitioned according to age threshold of 65 years. Horizontal lines represent the 95% sensitivity thresholds.

## Abbreviations

CPGs, clinical practice guidelines; PCa, prostatic cancer; PSA, prostate-specific antigen; PPV, positive predictive value; ISUP, International Society of Urological Pathology; AIC, Akaike information criterion; AUC, area under the ROC curve; LOWESS, locally weighted scatterplot smoothing; CI, 95% confidence interval; OR, odds ratio; NPV, negative predictive value; 4Kscore, 4-kallikrein panel score.
