# Supplementary material for: Definition of Outcome-Based Prostate-Specific Antigen (PSA) Thresholds for Advanced Prostate Cancer Risk Prediction
Source: Cancers (Basel). 2021 Jul 6;13(14):3381. doi: 10.3390/cancers13143381 (PMC8305281; doi:10.3390/cancers13143381)
Supplement: Supplementary file 1 [file cancers-13-03381-s001.zip › cancers-1227569-supplementary.pdf]

cancers

MDPI

Article

Definition of Outcome-Based Prostate-Specific Antigen (PSA) Thresholds for Advanced Prostate Cancer Risk Prediction

Simona Ferraro, Marco Bussetti, Niccolò Bassani, Roberta Simona Rossi, Giacomo Piero Incarbone, Filippo Bianchi, Marco Maggioni, Letterio Runza, Ferruccio Ceriotti and Mauro Panteghini

Supplementary Materials:

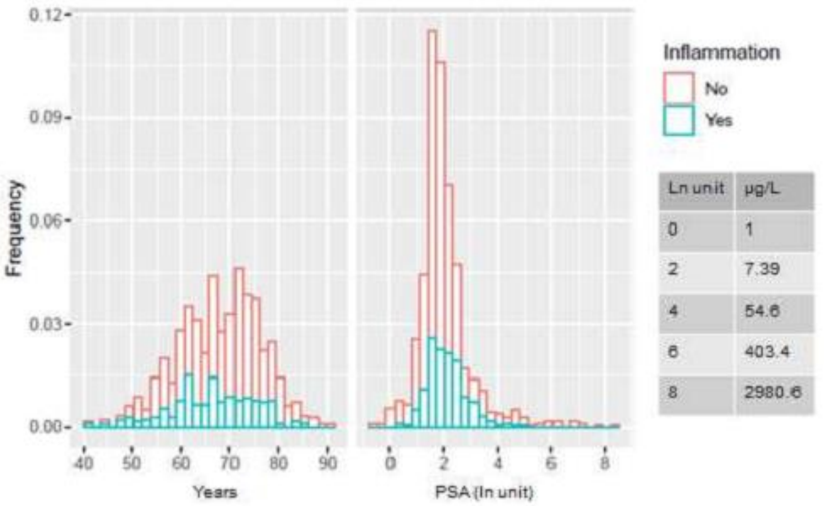

Figure S1. Distribution of age (left) and ln PSA concentrations (right) in patients patients with and without histological evidence of glandular inflammation.

Citation: Ferraro, S.; Bussetti, M.; Bassani, N.; Rossi, R.S.; Incarbone, G.P.; Bianchi, F.; Maggioni, M.; Runza, L.; Ceriotti, F.; Panteghini, M. Definition of Outcome-Based Prostate-Specific Antigen (PSA) Thresholds for Advanced Prostate Cancer Risk Prediction. *Cancers* **2021**, *13*, 3381. https://doi.org/10.3390/cancers13143381

Academic Editor: Delila Gasi Tandefelt

Received: 3 May 2021
Accepted: 30 June 2021
Published: 6 July 2021

Publisher's Note: MDPI stays neutral with regard to jurisdictional claims in published maps and institutional affiliations.

Copyright: © 2021 by the authors. Submitted for possible open access publication under the terms and conditions of the Creative Commons Attribution (CC BY) license (http://creativecommons.org/licenses/by/4.0/).

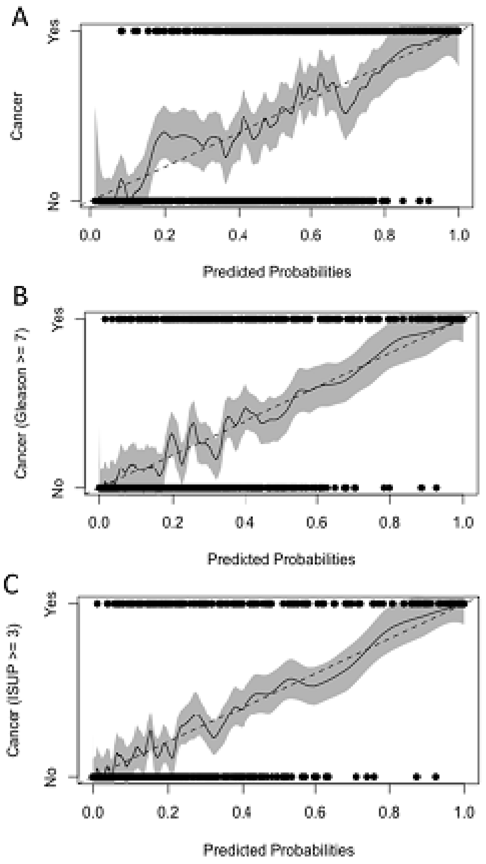

Figure S2. Calibration plots reporting the predicted probability of cancer of any grade (A), Gleason's score ≥ 7 (B) and ISUP grade ≥ 3 (C) cancers by considering the overall case series. The grey zone represents the variability of the predicted values around the bisector. The cases are reported as black points.

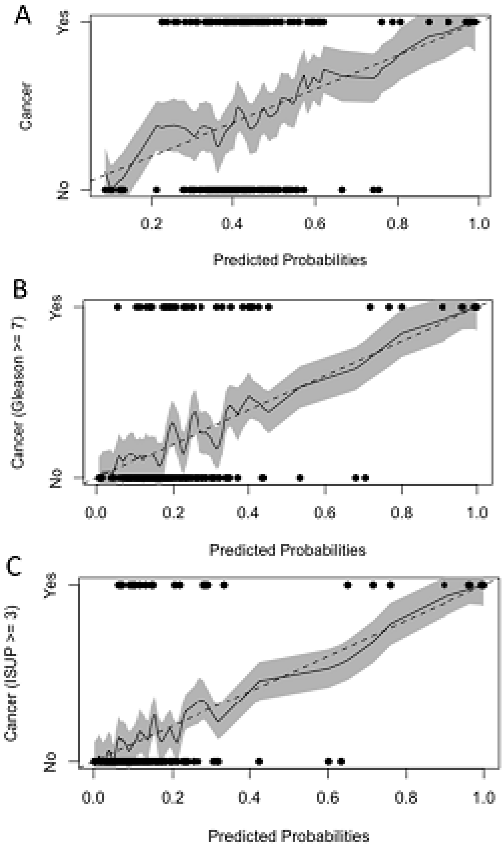

Figure S3. Calibration plots reporting the predicted probability of cancer of any grade (A), Gleason's score ≥ 7 (B) and ISUP grade ≥ 3 (C) cancers by considering <65 years old patients with no histological evidence of glandular inflammation. The grey zone represents the variability of the predicted values around the bisector. The cases are reported as black points.

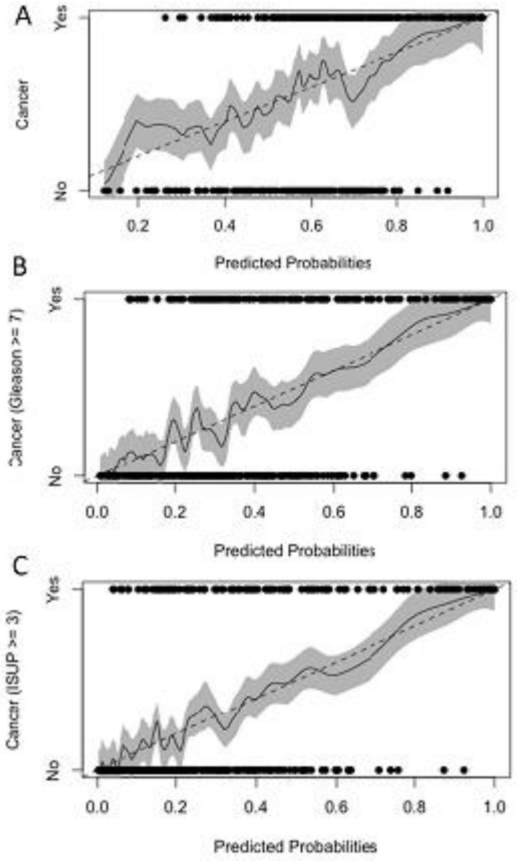

Figure S4. Calibration plots reporting the predicted probability of cancer of any grade (A), Gleason's score ≥ 7 (B) and ISUP grade ≥ 3 (C) cancers by considering ≥65 years old patients with no histological evidence of glandular inflammation. The grey zone represents the variability of the predicted values around the bisector. The cases are reported as black points.

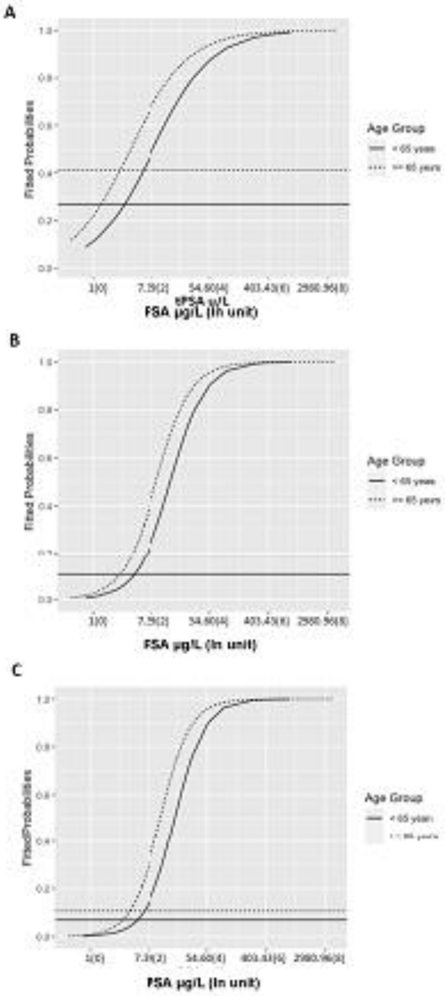

Figure S5. Fitted probabilities of prostatic cancer of any grade (A), with Gleason's score ≥ 7 (B), and ISUP grade ≥ 3 (C) vs. PSA values (ln scale units) in the subgroup of patients with no histological evidence of glandular inflammation, partitioned according to age threshold of 65 years. Horizontal lines represent the 95% sensitivity thresholds.

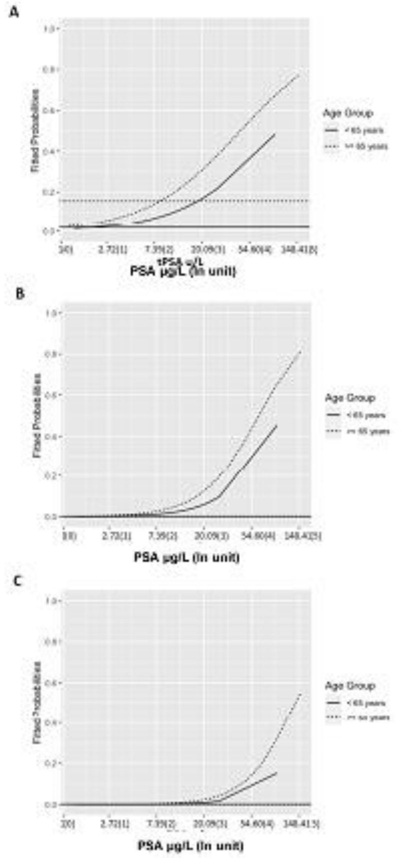

Figure S6. Fitted probabilities of prostatic cancer of any grade (A), with Gleason's score ≥ 7 (B), and ISUP grade ≥ 3 (C) vs. PSA values (ln scale units) in the subgroup of patients with glandular inflammation, partitioned according to age threshold of 65 years. Horizontal lines represent the 95% sensitivity thresholds.
